# 1112. Vancomycin Nephrotoxicity Relative to Alternative Antibiotic Treatments: A Systematic Review and Meta-Analysis of Randomized Controlled Trials

**DOI:** 10.1093/ofid/ofab466.1306

**Published:** 2021-12-04

**Authors:** Nayle Ibragimova, Katherine Huynh, Vanessa Ng, Vionna Wong, Evan J Zasowski

**Affiliations:** 1 Touro University California, Stockton, California; 2 Touro University CA, San Jose, California; 3 Tuoro University California, Vallejo, CA

## Abstract

**Background:**

Vancomycin is one of the most frequently prescribed antibiotics. Existing clinical evidence on vancomycin nephrotoxicity is limited to observational studies which are prone to confounding and bias. The purpose of this systematic review and meta-analysis is to compare acute kidney injury between vancomycin and comparator anti-methicillin resistant *Staphylococcus aureus* (MRSA) antibiotics using randomized controlled trial (RCT) data.

**Methods:**

PubMed and Embase were searched for RCTs comparing intravenous vancomycin to other anti-MRSA antibiotics in adult patients, published from 1990 to January 2021. Studies were included if they reported comparative data on renal outcomes. The primary outcome was change in renal function, referred to as ‘nephrotoxicity’ in this study. Studies where another known nephrotoxic medication was part of study therapy in any treatment group were excluded. Eighteen studies met the inclusion criteria, and two independent reviewers assessed the risk of bias. Data on nephrotoxicity definition, comparator drug, infection type, vancomycin dosing strategy, duration of treatment, and concurrent gram-negative coverage were extracted. Odds ratios (ORs) with 95% confidence intervals (CIs) were calculated.

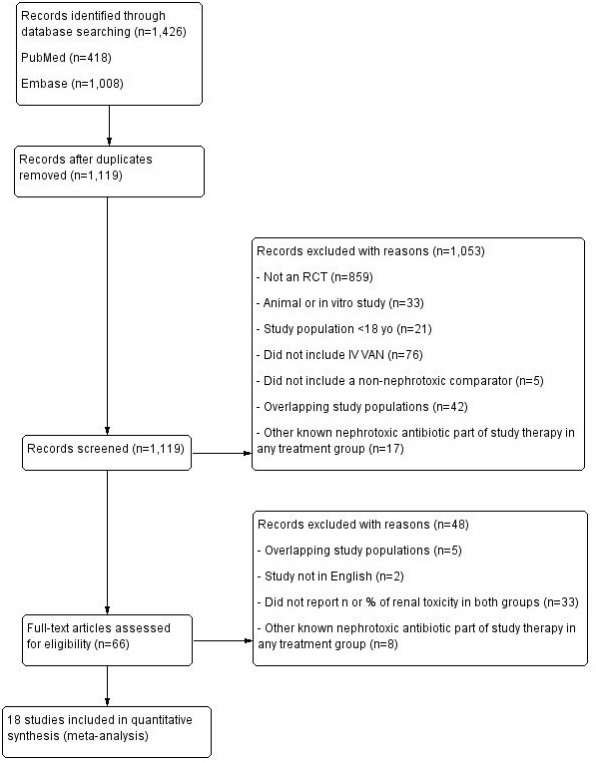

Figure 1. Flow chart of article selection.

**Results:**

Of 1,426 studies identified, 18 encompassing 8,966 patients were included. Treatment with vancomycin was associated with significantly increased odds of nephrotoxicity (OR, 2.41; 95% CI, 1.71 to 3.40; P< 0.00001) relative to its alternatives. A subgroup analysis grouping studies by reported vancomycin dosing approach revealed a stronger association between vancomycin and nephrotoxicity in studies with fixed-dose vancomycin regimens (OR 5.31; 95% CI 1.93 to 14.56; P=0.001) relative to studies with vancomycin therapeutic drug monitoring (TDM) (OR 2.17; 95% CI 1.51 to 3.13; P< 0.0001).

Figure 2. Forest plot indicating the risk of nephrotoxicity associated with vancomycin vs. comparators.

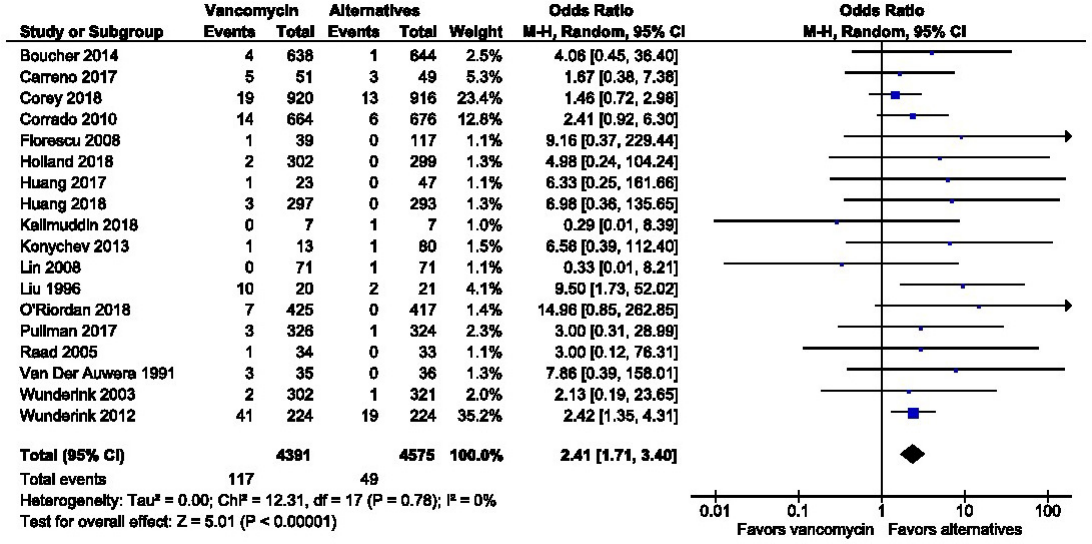

Odds ratios (ORs) and 95% confidence intervals (95% CIs) are shown for each study and the pooled analysis using a random effects model and the Mantel-Haenszel method. OR>1 means that the risk of kidney injury in the vancomycin group is greater than that in the comparator group.

Figure 3. Forest plot indicating a strong association between vancomycin and nephrotoxicity in studies with fixed-dose vancomycin regimens relative to studies with vancomycin therapeutic drug monitoring (TDM).

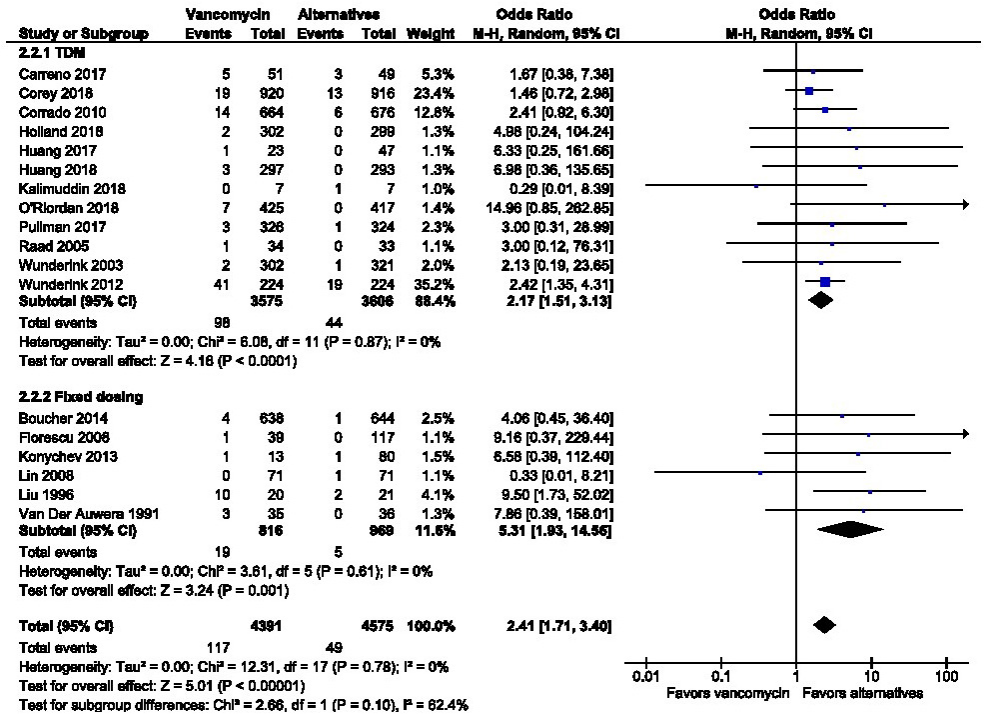

Odds ratios (ORs) and 95% confidence intervals (95% CIs) are shown for each study and the pooled analysis using a random effects model and the Mantel-Haenszel method. OR>1 means that the risk of kidney injury in the vancomycin group is greater than that in the comparator group.

Table 1. Summary of included studies.

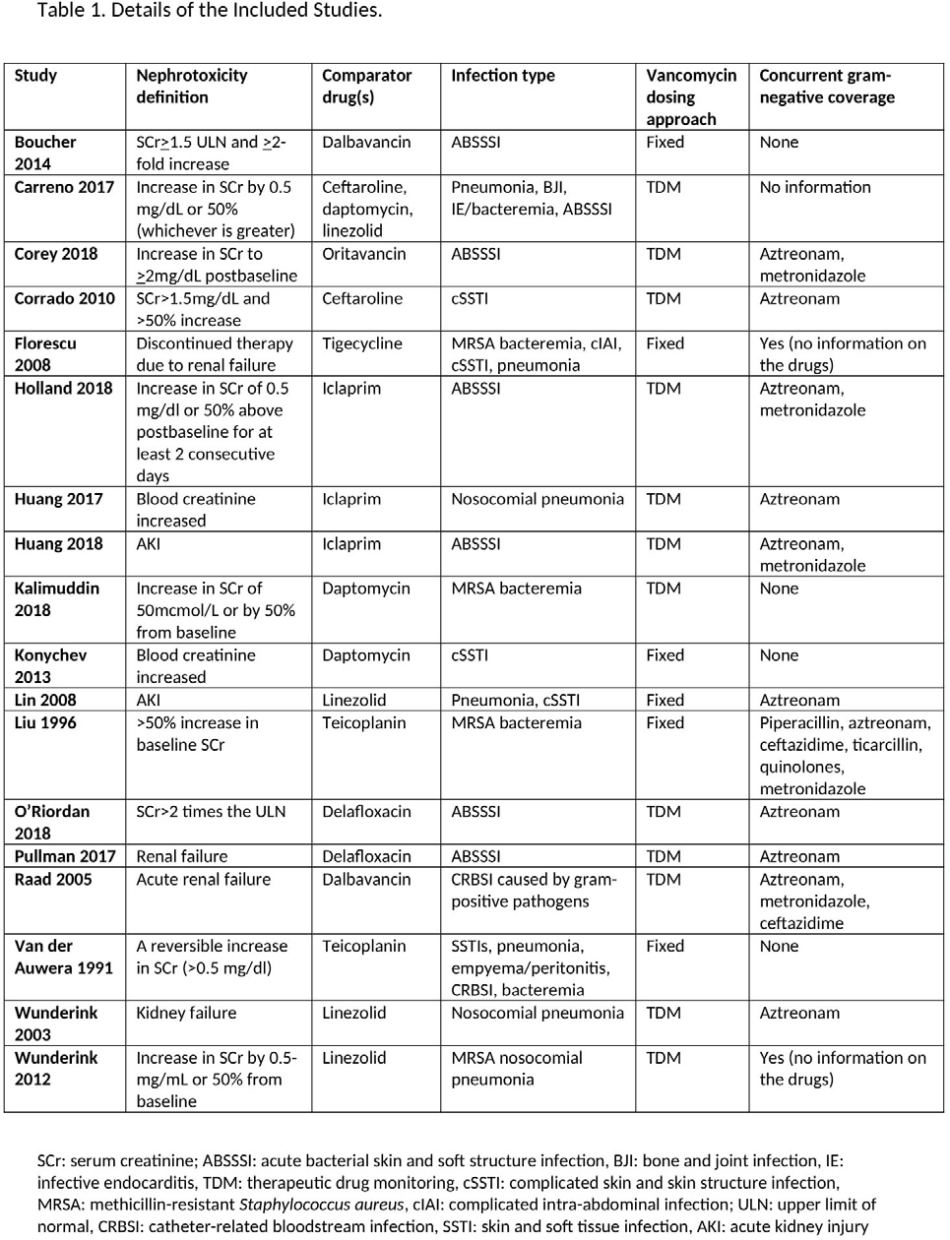

**Conclusion:**

This analysis shows that intravenous vancomycin is associated with greater odds of renal toxicity relative to alternative antibiotics. This effect was not as pronounced in studies where vancomycin TDM was used potentially indicating benefit of TDM although further study is required to confirm this.

**Disclosures:**

**All Authors**: No reported disclosures

